# Shear stiffening gel-enabled twisted string for bio-inspired robot actuators

**DOI:** 10.1038/s41598-024-55405-x

**Published:** 2024-02-27

**Authors:** Qingqing Zhang, Yuxuan Xue, Yafei Zhao, Kehan Zou, Wenbo Yuan, Yuqing Tian, Jiaming Chen, Jiangcheng Chen, Ning Xi

**Affiliations:** Department of Industrial and Manufacturing System Engineering, The University of Hong Kong, Hong Kong SAR, China

**Keywords:** Mechanical engineering, Biomaterials, Soft materials

## Abstract

A rotary motor combined with fibrous string demonstrates excellent performance because it is powerful, lightweight, and prone to large strokes; however, the stiffness range and force-generating capability of twisted string transmission systems are limited. Here, we present a variable stiffness artificial muscle generated by impregnating shear stiffening gels (STGs) into a twisted string actuator (TSA). A high twisting speed produces a large impact force and causes shear stiffening of the STG, thereby improving the elasticity, stiffness, force capacity, and response time of the TSA. We show that at a twisting speed of 4186 rpm, the elasticity of an STG-TSA reached 30.92 N/mm, whereas at a low twisting speed of 200 rpm, it was only 10.51 N/mm. In addition, the STG-TSA exhibited a more prominent shear stiffening effect under a high stiffness load. Our work provides a promising approach for artificial muscles to coactivate with human muscles to effectively compensate for motion.

## Introduction

In the growing fields of wearable assistive robots, prosthetics, and rehabilitation devices, variable stiffness actuators are being exploited and implemented because of their advantages of both safe human–robot interactions and broad force-generating capabilities^1,2^. Traditional actuators are assumed to be as stiff as possible to ensure high assistive force and precise position movements; however, rigid elements can facilitate large inertia and misalignments, which lead to unnatural motion patterns, discomfort, and, in some extreme cases, articular injuries^3^. Soft actuators such as shape memory polymers (SMPs)^4^ and pneumatic muscle actuators (PMAs)^5^, which actuate without complex linkages and have inherent or structural compliance, can produce complex bending, twisting, and folding motions; however, the torque capacity is restricted, and the requirement of external equipment for temperature or fluid supply makes portability a major challenge in terms of implementation^6^. In particular, artificial muscle made from fishing lines can achieve a 24-fold increase in tensile modulus but requires a temperature reaching 150 °C ^7^. Pneumatic particle jamming can achieve a reversible transition between a fluid-like state and a solid-like state by removing the vacuum from membranes filled with granules^8–10^. Elastomers coupled with magnetic particles under the action of a magnetic or electric field can change their rheological properties and possess a tunable stiffness capability^11^. However, actuators of this nature rely on additional equipment, limiting the mobility of the integrated system.

**Figure 1:**
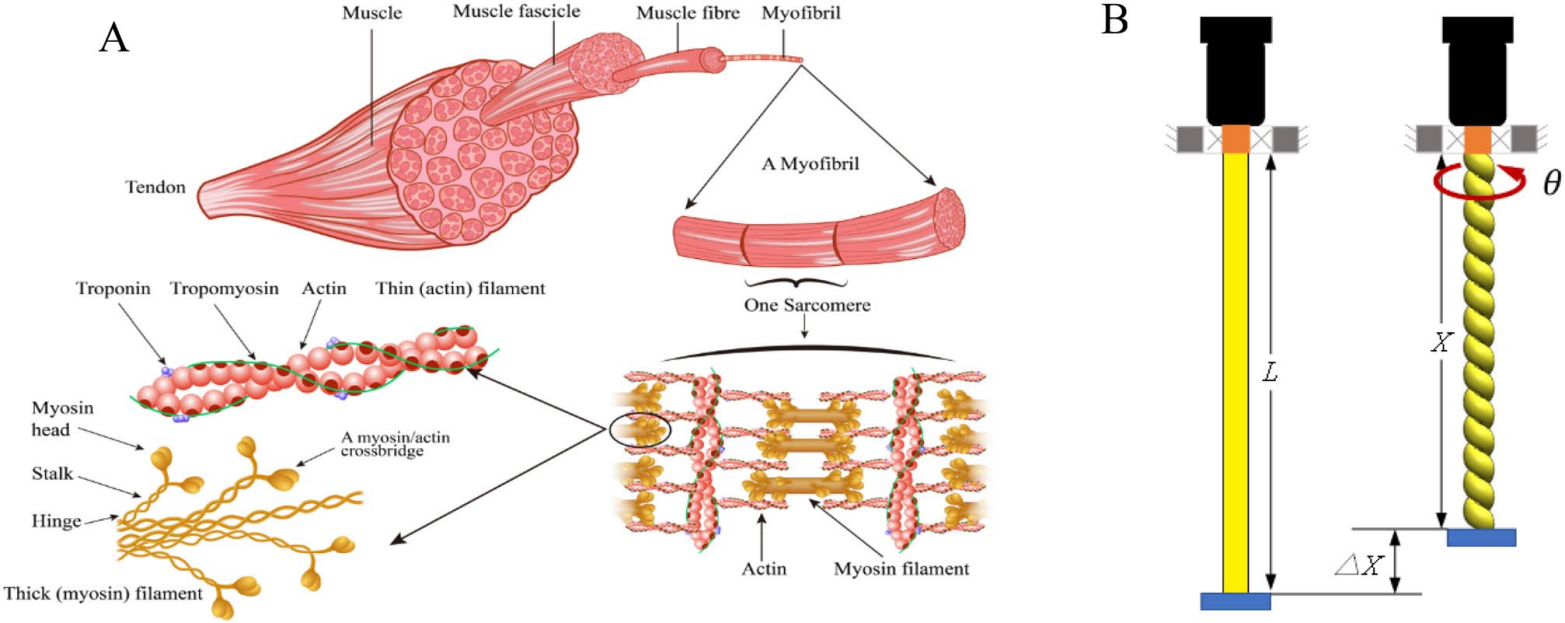
Structure of human muscle and the twisted string actuator (TSA). (**A**) The structure of human skeletal muscle. The stiffness and force generated by muscles depend on the number of activated cross-bridges. (**B**) The driving principle of TSA is to convert the torsional angle into linear displacement, thereby generating a pulling force on the load.

Human muscles provide inspiration in terms of stiffness modulation; for example, the muscle stiffness in a human arm can be tuned to adapt to different conditions, e.g., holding chopsticks or lifting heavy loads. Figure 1A shows the structure of a skeletal muscle, which holds thousands of muscle fibres. Each fibre consists of many myofibrils that are instead composed of thread-like sarcomeres (the basic contractile unit). Thin actin and the thick myosin filaments of sarcomeres are the two main active structures responsible for generating forces by forming cross-bridges between the heads of myosin molecules and adjacent actin molecules. The stiffness of muscle increases linearly under tension because of the parallel organization of cross-bridges and the increase in the number of activated cross-bridges^12^. A motor unit contains all the muscle fibres that are innervated by one motor neuron, and all the fibres contract when the motor unit is activated. A single motor unit in human toe extensors stimulated in a large frequency range can increase stiffness by approximately five times from rest to peak twitch^13,14^. Furthermore, a 50-fold stiffness change in a human ankle due to different levels of triceps surae activation has been measured^12^.

Wearable robot actuators allow motion assistance based on the intention of the user through myoelectric activation. Indeed, muscle-like actuators with variable stiffnesses are desirable for coactivation with human muscles to produce compensatory motion^15,16^. In this paper, inspired by human muscles in terms of stiffness tuning and manner of contracting, a new type of variable stiffness actuator is explored by combining a twisted string actuator (TSA) with shear stiffening gels (STGs), producing biologically inspired motions and matching the broad stiffness performance of skeletal muscles.

## Materials and fabrication

Twisted string actuators (TSAs) convert the rotary motion of a motor into linear motion by twisting the strings^17–20,35^ (Fig. 1B). Textile fibres with a braided structure and high surface friction were selected so that STG could be coated firmly on their surface. A monofilament string with a smooth surface, such as nylon, is not satisfactory. The braided Kevlar rope and Dyneema lines exhibit excellent performance in terms of high flexibility, large modulus, high strength, and low density (Supplementary Table 1). These materials are widely applied in soft armour that is integrated with STG^21,22^ and used for actuation of TSAs^15,19^. Therefore, braided Kevlar ropes and Dyneema fibres were chosen for fabricating an STG-based TSA.

The STG is strain-rate dependent and can display reversible variable stiffness without additional stimuli, such as external electric or magnetic fields. In its normal state, STG is soft and viscous with great self-healing ability. When shear, compression, and tension impact forces are applied, the strain rate increases, and the elastic modulus, storage modulus, and yield stress are critically enhanced^23–25^. The selected STG component was polyborondimethylsiloxane (PBDMS), which is more stable and has a higher initial viscosity than traditional shear thickening fluids (STFs) composed of nanosilica and polyethylene glycol. The storage modulus of PBDMS exhibits an increase of 2 orders of magnitude with increasing strain rate (Supplementary Fig. 1).

**Figure 2:**
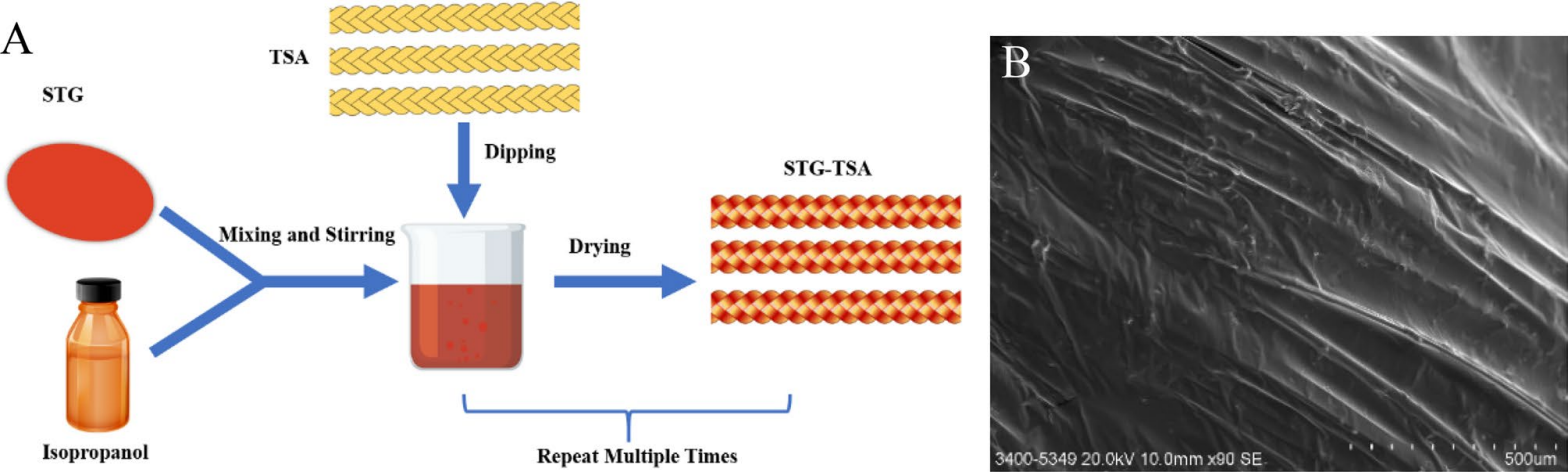
Fabrication and characterization of STG-TSA. (**A**) The “dip and dry” fabrication process of STG-TSA. (**B**) SEM image of a string coated with STG; scale bar: 500.0 µm.

The braided Kevlar ropes and Dyneema lines were dropped into the suspension, which was a uniform mixture formed by dissolving STG in an isopropanol solution, after which the strings with sticky STG were vulcanized in an oven. The effective “soak and dry” method^22,25^ was repeated many times to ensure that a sufficient amount of STG adheres uniformly to the strings (Fig. 2A). Scanning electron microscopy (SEM) images were captured at various magnification levels for both the Dyneema line and the STG-Dyneema line. In contrast to the SEM images of Dyneema (Supplementary Figs. 2C and D), the SEM images of STG-Dyneema exhibited a well-integrated structure with the strings, effectively filling the gaps between the filaments (Fig. 2B and Supplementary Fig. 2A).

## Results and discussion

### Nanomechanical characterization of STG based on AFM

**Figure 3:**
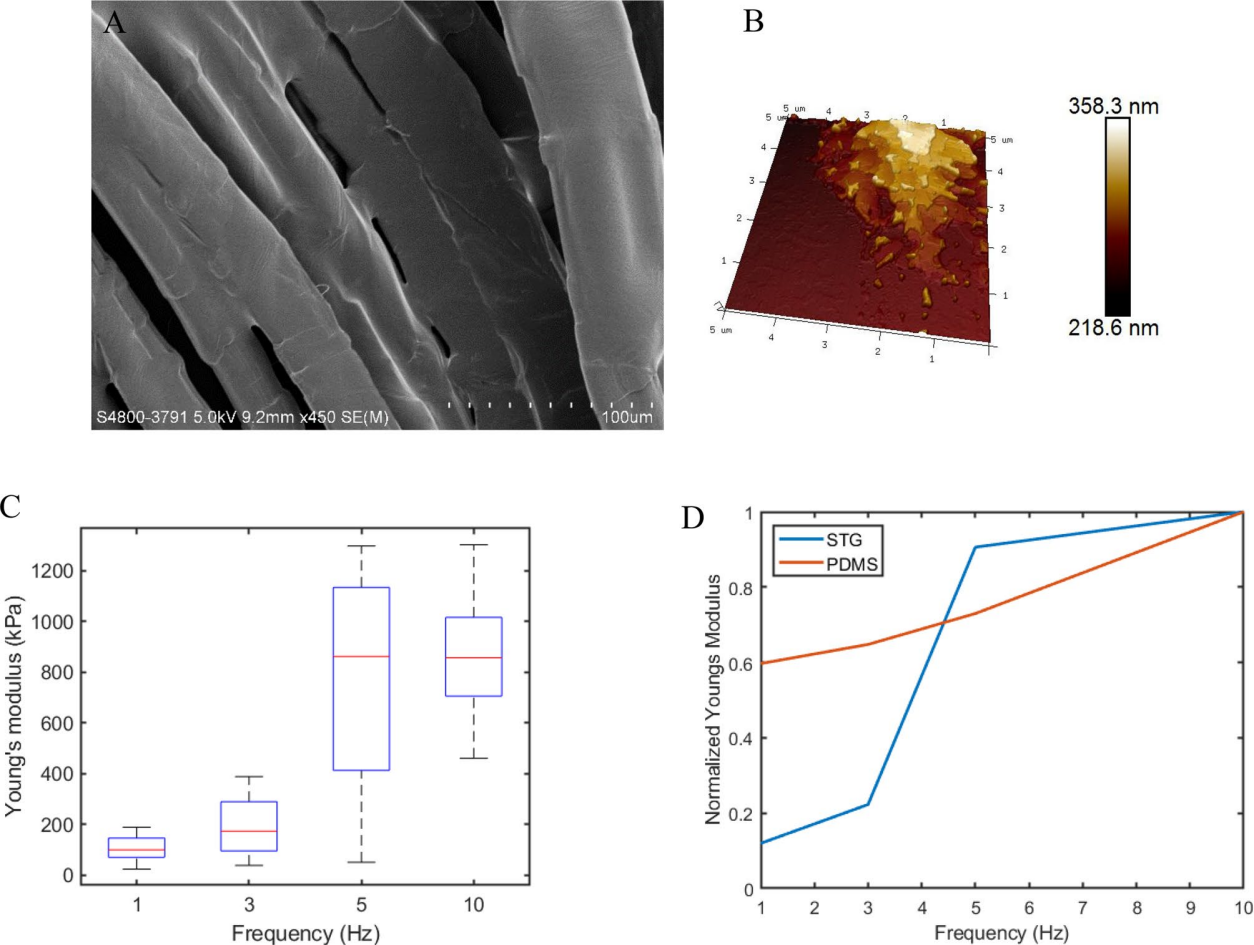
Nanomechanical properties of STG based on AFM. (**A**) The distribution of thin-film STGs within fibres; scale bar: 100.0 µm. (**B**) AFM topography of STG in a thin-film state. The thickness of STG coated on cleaved mica is consistent with the state of STG adhering to the fibres; scan size: 5 µm X 5 µm. (**C**) Young’s modulus distributions of the STG at different indentation frequencies; the medians are 99.48 kPa, 173.02 kPa, 861.54 kPa, and 864.06 kPa at frequencies of 1 Hz, 3 Hz, 5 Hz, and 10 Hz, respectively. (**D**) Normalized Young’s modulus of STG and PDMS at different indentation frequencies.

The mechanical properties of STG are usually characterized via a rheometer, using a sample with a thickness of a few millimetres and a diameter of 20 mm^21^ (Supplementary Fig. 1); the morphology of this sample differs from that of STG when coated on fibres. Since STG is in a thin-film state with a microscale thickness, the mechanical properties may exhibit different behaviours when subjected to compression and shear forces during twisting. No experiments have previously been conducted to test the strain-rate stiffening property of STG on the microstructure; therefore, atomic force microscopy (AFM) was adopted to evaluate the stiffness variation at different indentation frequencies^26,27^. A thin coating of STG on cleaved mica with a thickness consistent with the state of STG coated on the fibres (Fig. 3A) was prepared for the AFM measurements. The topography of the nanoscale-film STG was determined in PeakForce tapping mode, and the height distributions at 5 μm × 5 μm and 1 µm × 1 µm are presented in Fig. 3B and Supplementary Fig. 3A. Figure 3C shows the distributions of Young’s modulus in the thin-film STG under different indentation frequencies, and the probabilistic density function of Young's modulus of the STG at these frequencies is presented in Supplementary Fig. 5. The results indicate that the thin-film STG exhibits a typical strain-rate stiffening characteristic, in which the medians and the mean values (shown in Supplementary Fig. 6A) of Young’s modulus increase with increasing indentation frequency. However, the critical indentation frequency needed to cause a stiffening effect is 5 Hz, which is higher than the critical shear frequency from the rheometer results.

PDMS exhibits typical hyperelastic behaviour that is independent of the strain rate applied in a certain strain range^28,29^. A standard sample was selected as the control group for a comparison of the strain-rate stiffening effect of STG. The topography of the PDMS sample is shown in Supplementary Fig. 3B. The results showed that the mean Young’s modulus values of the PDMS films were 4.08 ± 0.22 MPa, 4.43 ± 0.24 MPa, 4.99 ± 0.32 MPa, and 6.83 ± 0.35 MPa at frequencies of 1 Hz, 3 Hz, 5 Hz, and 10 Hz, respectively (Supplementary Figs. 6B and 16). After normalizing the Young’s moduli of STG and PDMS separately according to the value at 10 Hz (the maximum of Young's modulus in the four frequencies) (Fig. 3D), the results revealed that the stiffness of PDMS was almost rate independent, but STG was able to achieve a wide range of stiffnesses by tuning the strain rate. The strain-rate stiffening property of STG makes it suitable for soft robotics, where a large range of stiffnesses is desired.

### Mechanical performance of STG-TSA

**Figure 4:**
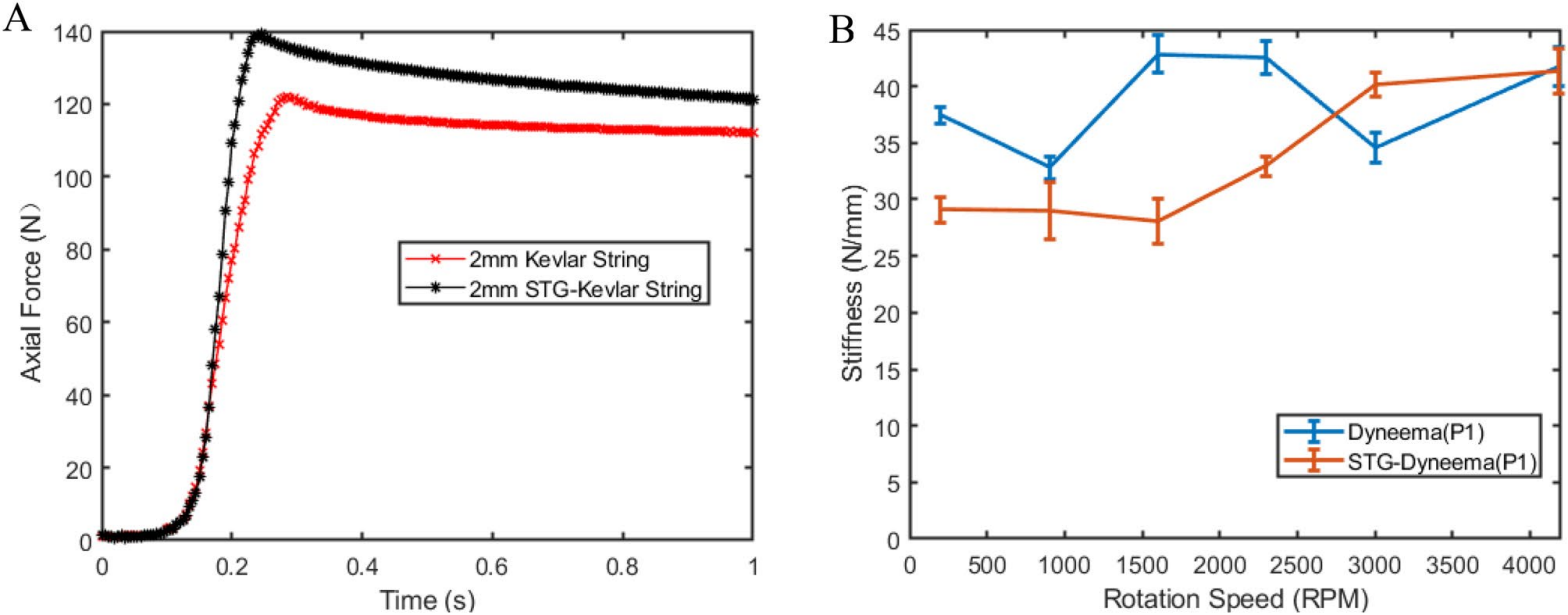
Shear stiffening effect of STG-TSA. (**A**) Comparison of the generated pulling force and contraction time between the Kevlar rope and STG-Kevlar rope with a 2 mm diameter and a 200 mm length. (**B**) Comparison of the axial stiffness (slope of linear fitting for axial force versus axial displacement) at different rotation speeds between the Dyneema line and STG-Dyneema line.

To observe the shear stiffening effect of the strings impregnated with STG, high-speed twisting tests were carried out on neat Kevlar ropes and STG-impregnated Kevlar ropes (STG-Kevlar). The detailed experimental settings are presented in Supplementary Table 4. Supplementary Figs. 17 and 18 illustrate the changes in speed and rotation as a function of pulling force. When a string was twisted to its maximum, the pulling force of the STG-Kevlar rope increased by almost 20 N compared to that of the neat Kevlar rope (Fig. 4A). This is because when twisting the string, the STG impregnated into the rope was sheared and compressed between the turns and filaments of the string. At the moment the string was twisted to its maximum, the compression and shear forces applied on the string increased abruptly, resulting in high strain rates acting on the STG. Subsequently, the shear stiffening effect occurred as the STG transitioned from a vicious state to an elastic state, and the stiffness of the STG-Kevlar rope increased, leading to the high transmission efficiency of the string to generate a larger pulling force.

The response time for assistant actuators is imperative because a robot will perturb muscle function and reduce the assistance level rather than provide support if the actuators are not synchronous with muscle contraction. The response time of the STG-Kevlar rope was improved by approximately 50 ms compared with that of the neat Kevlar rope (Fig. 4A); this is closer to the twitch time of muscles located in the lower leg (100 ms)^30^.

Strain-rate-dependent characteristics of STG–TSA were measured in velocity control mode at different rotation speeds. Both the Dyneema lines and the STG-Dyneema lines exhibited an increase in peak force and a reduction in response time with increasing twisting speed. All of the peak forces for STG-Dyneema were greater than those for Dyneema because of the presence of the elastic element STG, and they were affected by twisting speeds due to the increase in the storage modulus of STG with increasing strain rate. At a low twisting speed of 200 rpm to 2300 rpm, the peak force of STG-Dyneema did not show any obvious enhancement compared to that of Dyneema, and the response time of STG-Dyneema was even longer than that of Dyneema at 200 rpm and 900 rpm. However, when the rotation speed was greater than 3000 rpm, the peak pulling force of STG-Dyneema increased by approximately 13 N compared to that of Dyneema. In particular, the pulling force for STG-Dyneema increased from 6 to 12 N when the rotation speed increased from 2300 to 3000 rpm (Supplementary Fig. 9).

The axial stiffness, which is derived from the axial force $F_{Z}$ measured by the force sensor and the contraction Δ*X* calculated from the laser displacement sensor, exhibits different behaviours for STG-Dyneema and Dyneema. To assess the axial stiffness, a linear regression analysis was conducted on the axial force versus axial displacement data obtained at different rotation speeds. The slope of the linear fit represents the axial stiffness of the twisted string actuators, as demonstrated in Supplementary Fig. 14. In addition, the average value and standard deviation of the slopes from three trials at each rotation speed were depicted as error bars in Fig. 4B. The results indicate that the axial force-axial displacement slope of the STG-Dyneema actuators increased from 29 to 41 N/mm with increasing twisting speed. In contrast, the axial stiffness of Dyneema remained relatively constant at approximately 38 N/mm across different twisting speeds.

The fundamental reason for the axial stiffness variation under different rotation speeds when STG was introduced into the strings is the elasticity property derived from the tension–elongation relationships. Based on the helical geometry formed during twisting^17,18^ (the schematic depiction is shown in Supplementary Fig. 10), the string length $L$ under load conditions during twisting can be calculated from the motor angle $\theta$ recorded from the encoder and the contracted length $X$ measured by the laser displacement sensor; then, the elongation $\Delta L$ of the string can be described as:

1 $$\Delta L = L - L_{0} = \sqrt {\theta^{2} + X^{2} } - L_{0}$$

where *L*_0_ is the unloaded length.

The tension $F_{i}$ of the string can be derived from the axial force $F_{Z}$ and the formed helical angle *α* calculated from the formed right triangle as follows:

2 $$F_{i} = \frac{{F_{Z} }}{\cos \alpha } = \frac{{F_{Z} L}}{X}$$

**Figure 5:**
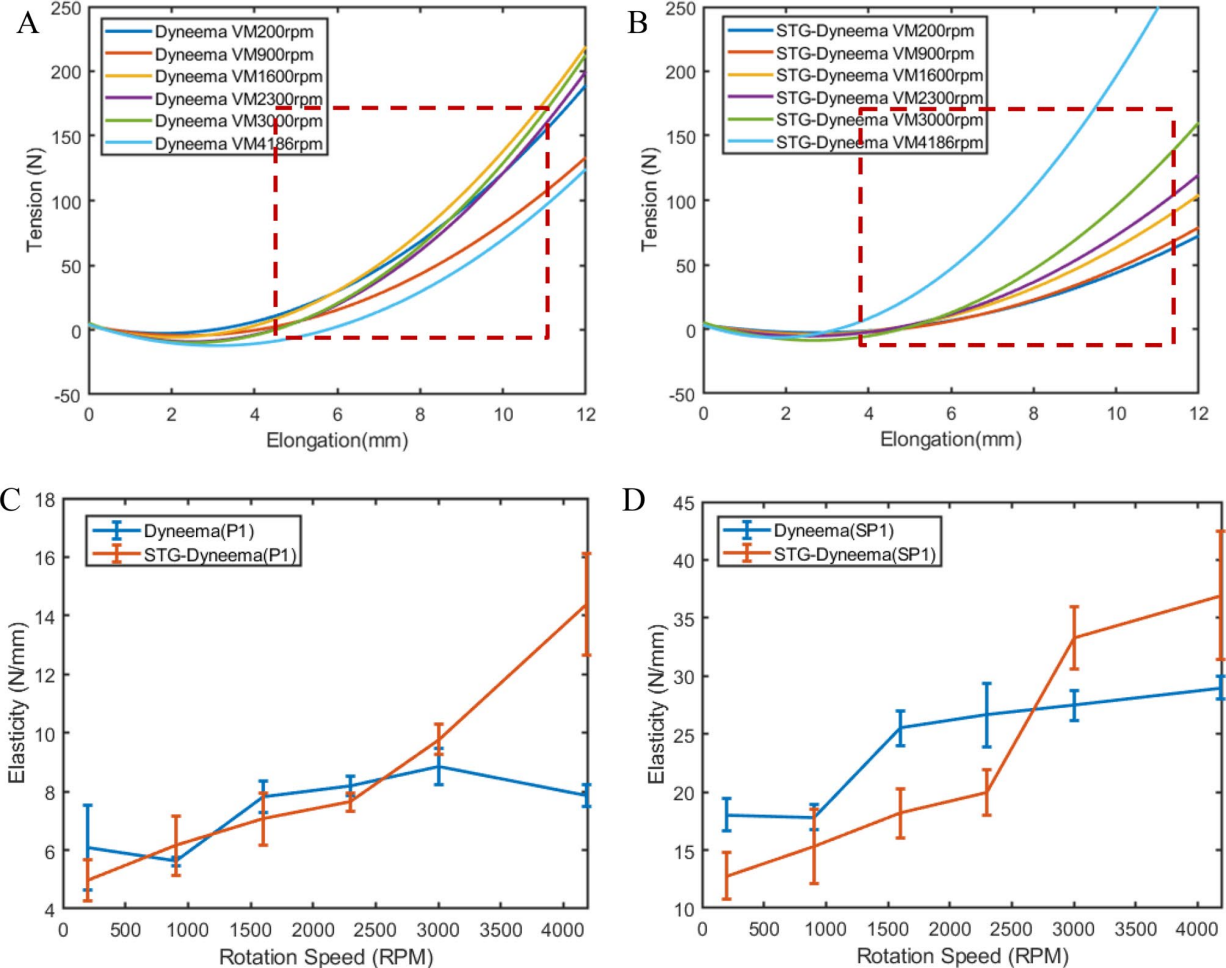
Strain rate-dependent characteristics of STG-TSA. Quadratic fitting of the derived tension versus elongation for (**A**) the Dyneema line and (**B**) the STG-Dyneema line at different rotation speeds (red dotted rectangular areas: steep region of the tension–elongation curves). (**C**) Comparison of the slopes of linear fitting for tension–elongation relationships at different rotation speeds between the Dyneema line and STG-Dyneema line. (**D**) Comparison of the elasticity (slope of linear fitting for a steep range of tension–elongation relationships) at different rotation speeds between the Dyneema line and STG-Dyneema line.

Supplementary Fig. 11A, B depict the relationship between the calculated string tension $F_{i}$ and calculated string elongation $\Delta L$ for both the Dyneema line and STG-Dyneema line in a set of experiments conducted at different rotation speeds. The fitted data from these figures are presented in Fig. 5A, B. Based on three sets of trials at each rotation speed, the average value and standard deviation of the slopes of the tension–elongation, which represent the elasticity of the twisted string actuators, were presented as error bars in Fig. 5C. The elasticity of STG-Dyneema exhibited a different trend than that of Dyneema with increasing twisting speed. For the Dyneema line, the elasticity at different rotation speeds were very similar, with a maximum value of 7.4 N/mm. This indicates that the elasticity of Dyneema is independent of the twisting speed. In contrast, the slopes of STG-Dyneema clearly increased with increasing twisting speed, and the value at 4186 rpm was three times greater than that at 200 rpm. The maximum value at a twisting speed of 4186 rpm was 14.38 N/mm, which was greater than the peak value for Dyneema.

These trends in elasticity are reinforced by the selected data presented in Fig. 5D. As gaps existed in the fibres of the string, a dead zone was created at the beginning of the twisting. Data from the steep regions of the tension–elongation curves (indicated by the red dotted rectangle areas) are expanded in Fig. 5A, B after being linearly fitted. The derived slopes of these lines reflect the actual elasticity of the strings at different twisting speeds. The average value and standard deviation of three sets of trials at each rotation speed were calculated and depicted in Fig. 5D. The results indicate that, in comparison to STG-Dyneema, the elasticity of Dyneema does not exhibit a significant increase with increasing twisting speed, but the elasticity of STG-Dyneema shows a threefold increase. The slopes of these linear fitting lines (Supplementary Table 2) provide a measure of the stiffness sensitivity of STG-Dyneema to changes in strain rate and reflect the ability of the STG to increase the stiffness and force-generating capability of TSA by applying different twisting speeds.

**Figure 6:**
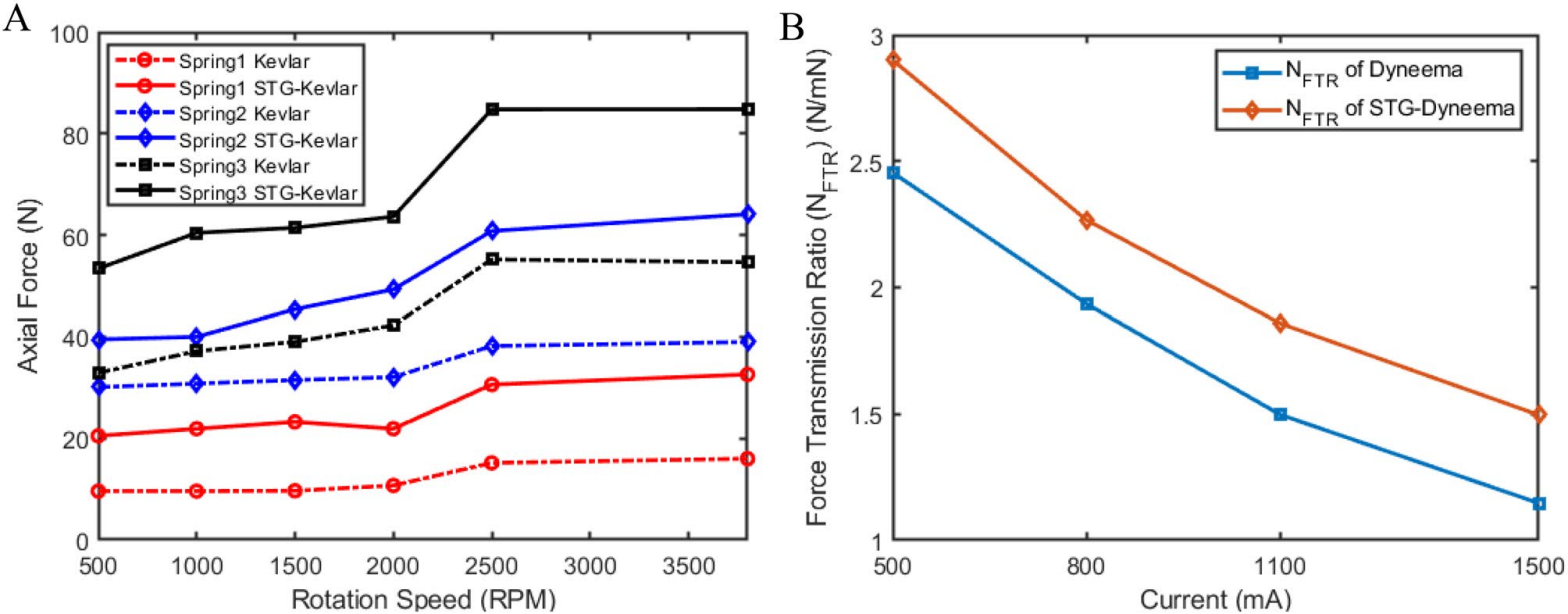
Performance evaluation of STG-TSA. (**A**) Peak forces at different rotation speeds for the Kevlar rope and STG-Kevlar rope with a 2 mm diameter and 300 mm length in load, with elastic coefficients of 0.9034 N/mm (spring 1), 4.4385 N/mm (spring 2), and 10.961 N/mm (spring 3). (**B**) Force transmission ratios of STG-Dyneema and Dyneema under different motor torque inputs.

We investigated the effects of load stiffness on the shear stiffening performance and force-generating capability of STG-TSA by introducing three springs with different elastic coefficients as the load (shown in Supplementary Table 3). The difference in the maximum force generated between STG-Kevlar and Kevlar increased with increasing load stiffness (Fig. 6A). At a twisting speed of 3800 rpm, the peak force of STG-Kevlar had a spring with the largest stiffness as the load increased by 30.15 N compared to that of Kevlar under the same load conditions. However, after applying a spring with the lowest stiffness as the load, the gap between the STG–Kevlar and Kevlar at a twisting speed of 3800 rpm was only 16.6 N. The greater the stiffness of the load was, the more significant the shear stiffening effect and the greater the force-generating capability of the STG-Kevlar. This is because a larger load caused a larger strain rate and subsequently enhanced the stiffness of STG–Kevlar. In addition, the increase in the peak force of STG-Kevlar relative to that of Kevlar improved with increasing twisting speed.

The force transmission ratios (FTRs)^20^ for the STG-TSA calculated by $N_{FTR} = F_{Z} /\tau_{in} \eta_{in}$  (*τ*_*in*_ is the input motor torque, and *η*_*in*_ is the transmission efficiency of the motor) are also impressive under different motor torque inputs. To further illustrate the excellent force transmission performance of STG-TSA, experiments were performed on both STG-TSA and TSA in current control mode. Both the maximum force and response time for STG-Dyneema were improved compared with those for Dyneema (Supplementary Fig. 15). We assumed that the motor had the same efficiency when the STG-Dyneema lines and Dyneema lines were twisted under the same current input. The FTRs were computed using the maximum force applied during twisting divided by the motor torque. Figure 6B presents the calculated FTRs of the STG-Dyneema lines and Dyneema lines under four current settings. Under the same motor torque input, STG-Dyneema always exhibited a greater FTR than Dyneema. The largest increase was 0.45. The introduction of STG greatly enhanced the force production capability of TSA.

### Comparisons to human muscles and applications

**Figure 7:**
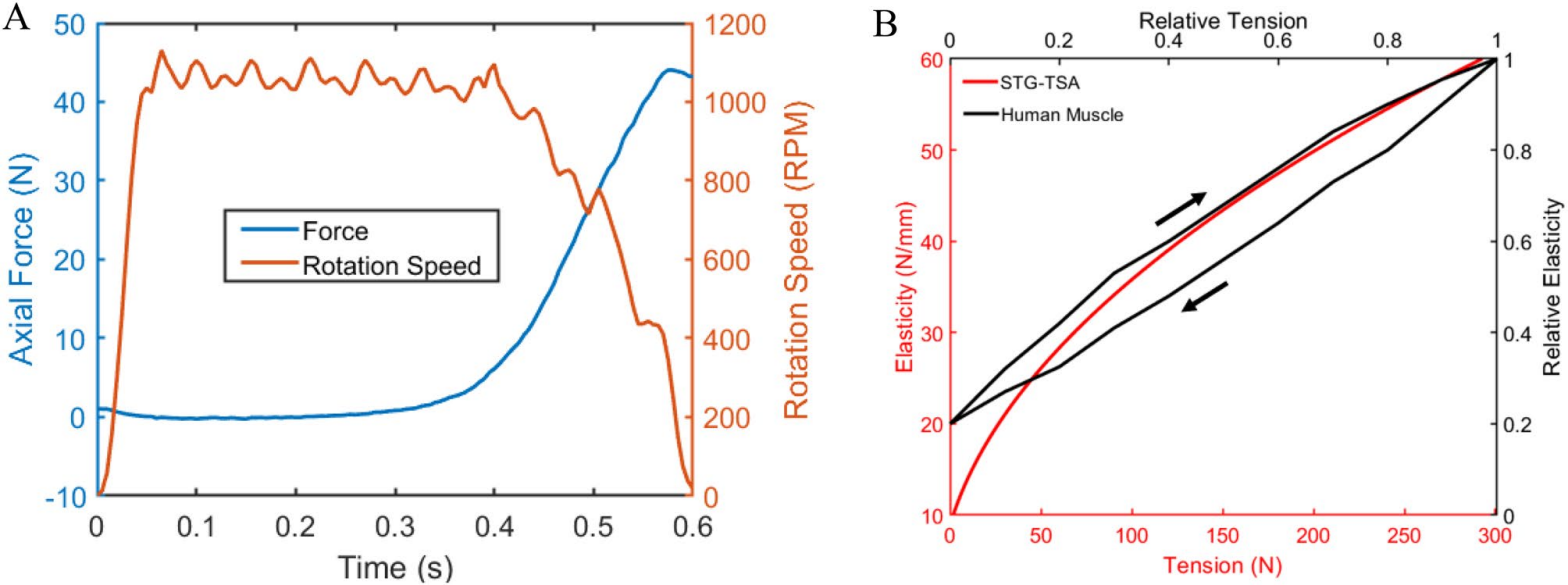
Comparison of characteristics between STG-TSA and human muscle. (**A**) The force–velocity relationship of the STG-TSA is highly similar to that of human muscles in concentric contraction. (**B**) Changes in elasticity with tension for the STG-TSA and human muscle.

The tension in human muscle increases when the shortening velocity decreases during concentric contraction because of cross-bridges breaking and reforming the contractile element, and viscosity is maintained in both the contractile element and peripheral tissue^30^. Figure 7A shows the force–velocity relationship of STG-TSA, where with decreasing rotation speed, the tension gradually increases, and the maximum tension is reached when the rotation speed is reduced to zero. Such a force–velocity characteristic highly corresponds with human muscle.

Single STG-TSA demonstrates the same mechanical properties as human muscle in terms of the elasticity–tension relationship and stiffness modulation. Figure 7B demonstrates that STG-TSA is similar to muscle in that with increasing tension, the elasticity gradually increases^12^. A single muscle fibre can reach a fivefold increase in stiffness resulting from a large range of activation frequencies^12,13^. For STG-TSA, the elasticity at a high rotation speed was almost 3 times greater than that at a low rotation speed, while this relationship was only 1.5 times greater for TSA (Fig. 5C). Thus, STG brings TSA closer to human muscle in terms of stiffness and tension regulation. In addition, variable stiffness tuning allows STG-TSA to assist human muscles and joints effectively. Inherent compliance enables flexibility and comfortable interactions, while stiffening is needed to output sufficient forces that are transferred to human joints when necessary.

In summary, we report a wearable soft actuator with a broad range of stiffnesses obtained by applying different twisting speeds (the significant improvements in performance of STG-TSA compared to TSA are summarized in Supplementary Table 5). We propose the “soak and dry” strategy, which incorporates the STG into the gap of the string to fabricate the STG-TSA effectively. To demonstrate the shear stiffening behaviour of thin-film STG coated on the fibres, we used AFM in PeakForce tapping mode to evaluate the stiffness variation at different indentation frequencies. Through high-speed twisting tests, we validated that the maximum pulling force and response time of the STG-TSA increased significantly. We demonstrated that the mechanical properties of STG-TSA are strongly dependent on the strain rate. The output force, axial stiffness, and elasticity of the STG-TSA increase with increasing twisting speed. In addition, we show that the STG-TSA has a more prominent shear-stiffening impact on loads with high stiffness, and the force capacity of the STG-TSA is enhanced remarkably in contrast to that of the TSA. This work provides opportunities for bioinspired robot actuators to imitate the nature of muscles, provide compensatory motion for joints, and cooperate and work simultaneously with humans, thereby creating hope for aged people, especially people with sarcopenia.

## Methods

### Fabrication of an STG-enabled twisted string

PBDMS is formed by introducing boric acid into PDMS^23,31–33^. Boric acid (AR degree, purchased from Sinopharm Chemical Reagent Co. Ltd., Shanghai, China) was mixed with hydroxyl silicone oil (AR degree, 500 mm^2^/s, purchased from Qingdao RZ-LABS Technology Co., Ltd., China) at 30 mg/ml in a beaker. The beaker was placed in an oil bath (LC-DF-101S), an electric mixer (LC-ES-200SH) was used to stir the solution at room temperature until homogeneous, the oil bath was heated slowly until the temperature reached 180 °C, and the mixture was stirred for 1 h at 180 °C. During the process, the silicone polymer mixture formed cross-links, becoming viscous and sticky. Then, STG was obtained when the polymer was cooled to room temperature.

Commercially available braided Kevlar ropes (purchased from Dongguan Haotai Special Rope Co., Ltd.) and Dyneema lines (purchased from Dongyang Fedu Fishing Tackle Limited Company) with diameters of 2 mm were chosen for the STG-based TSA. The mechanical properties of other commercial fibres used for muscle fabrication, including nylon and carbon fibres, are compared in Supplementary Table 1.

As reported by Wang, an effective “soak and dry” method was developed and improved upon to fabricate STG–TSA (Fig. 2A). First, Kevlar ropes and Dyneema lines were prepared with a 2 mm diameter and varying lengths. Second, a mass of 200 g of raw STG was prepared by diluting the STG with IP at a ratio of m (STG):m (IP) = 1:1 in a beaker for 1 h and stirring the mixture constantly until uniform. The strings were subsequently added to the suspension, which were then soaked thoroughly for 20 min. Finally, the strings with sticky STG were vulcanized in an oven at 50 °C for 30 min to remove the IP. The “soak and dry” steps were repeated many times until sufficient STG adhered to the fibres. Supplementary Fig. 7 shows the prepared STG-Dyneema lines and STG-Kevlar ropes.

The morphologies of the as-prepared STG-TSA samples were characterized by scanning electron microscopy (SEM; Hitachi S-3400N, and Hitachi S-4800), as shown in Fig. 2B and Supplementary Figs. 2A and B. The SEM images indicated that the STG integrated well with the string and filled the gaps between the filaments.

### Mechanical property test of STG

The shear stiffening effect of STG was measured using a commercial Anton Paar MCR 302 rheometer with a 20-mm-diameter parallel-plate geometry. The sample was cut into a coin shape with a diameter of 20 mm and a thickness of 1 mm. The dynamic oscillation shear mode was adopted, with a shear frequency swept from 0.628 to 628 rad/s and a strain amplitude set at 0.1%. Supplementary Fig. 1 shows the storage modulus and loss modulus of STG under dynamic oscillatory shear. The shear frequency varies from 10^–1^ to 10^2^ Hz, while the storage modulus increases from 4509 Pa to 0.25 MPa. The STG exhibits a typical shear stiffening effect and changes from a viscous state to an elastic state at a shear frequency of 1 Hz.

To further observe the nanomechanical properties of STG, AFM was used to determine the variation in Young’s modulus under different strain rates, and a standard PDMS sample (Bruker, Inc.) with a Young’s modulus of 3.5 MPa was selected as the control group. Raw STG with a mass of 3 g was prepared, diluted in isopropyl alcohol at a ratio of 15%, and stirred constantly until homogenous. Cleaved mica sheets were prepared for the experiment, and 20 μl of mixture medium was coated on the mica surface and gently air-dried. A thin layer of STG was formed on mica and used for topography imaging. A cone-shaped probe (ScanAsyst-air (Bruker)) with a typical spring constant of 0.4 N/m was applied to scan the specimens in the air. At least three samples were prepared, and scanning was maintained with PeakForce frequencies of 0.5 to 1 kHz to ensure reliability with a resolution of 256 × 256 pixels.

ForceVolume tapping mode was used to quantitatively characterize the elastic properties of STG at different strain rates^34^. According to the storage modulus tested using a rheometer, a DNP-10 probe (Bruker, Inc.) with an Au coating and a typical stiffness of 0.24 N/m was selected. The raw STG with a thickness of 5 µm was prepared and tiled flat on a mica sheet. Then, the mechanical properties of the thin-film STGs were tested at multiple locations via indentation experiments using PBS buffer. Supplementary Fig. 4A shows a schematic depiction of the indentation and retraction process when the probe applied force to the STG. The applied force of the probe was controlled from 2 to 4 nN. Applying different indentation frequencies when the cantilever approaches the surface of the specimens allows for the measurement of the elastic properties of STG at different strain rates. Supplementary Fig. 4B presents a typical force curve of the STG for a pulling cycle at a frequency of 1 Hz. Two hundred–300 sets of data were collected at each indentation frequency to reduce error and improve the accuracy of the experiments.

Since a cone-shaped probe was used in ForceVolume tapping mode and the opening angle was 15°, the Sneddon model was adopted to fit the approach part of the force curves to calculate Young’s modulus as follows:

3 $${\text{F }} = \, \frac{2}{\pi }\frac{{\text{E}}}{{{1} - {\text{n}}^{{2}} }}{\text{tan }}(\alpha )\delta^{{2}}$$

Here, *F* is the interaction force generated by the AFM probe, and the value is equal to the cantilever deflection times the spring constant. *δ* is the depth of indentation, and the value is the displacement of the piezoelectric actuator in the z direction minus the deflection of the cantilever. *α* is the half-angle of the conical tip. *ν* is Poisson’s ratio of STG with a typical value of 0.5. *ν* is the fitted Young’s modulus.

### Design of the experimental platform

To verify the strain-rate dependent characteristics and evaluate the performance of the proposed STG-TSA, we developed a testbed, as shown in Supplementary Fig. 8. The testbed consisted of a brushless DC micromotor (Type: 1215) equipped with a 1024 CPT encoder and a reducer with a reduction ratio of 3.75:1 (purchased from Shenzhen Xuandong Technology Co., Ltd.), a force sensor (ZNLBS-50KG), a laser displacement sensor (Panasonic HG-C1400), and a string. A force sensor was used to measure the axial actuation force, an encoder was used to measure the rotation angle, and the axial contraction of the string was recorded by the laser displacement sensor. The data acquired by the force sensor, laser displacement sensor, and encoder were collected by the NI PCIe-6321 DAQ card. A DC brushless servo driver (DUAL6010) was used to control the rotational speed and output torque, and velocity mode control and current mode control were implemented to control the STG-TSA.

As shown in Supplementary Fig. 8A, in the experiments involving two-end fixed conditions, the motor was mounted horizontally on a frame fixed on a guide rail, and one end of the string was connected to the output shaft of the motor by coupling. The other end of the string was connected to a special clamp jointed with the force sensor and then fixed to the bracket on the guide rail. The bracket was restricted by a stop collar in the motor axis direction. The vertical bearing and guide rail were fixed on two XYZ-axis moving platforms to adjust the position separately.

In the spring load experiments (shown in Supplementary Fig. 8B), the bracket on the guide rail was linked with a spring by a hook, and the other end of the spring was joined with a bracket that was fixed on another XYZ-axis moving platform, also by a hook.

### High-speed twisting tests

Three neat Kevlar ropes and three Kevlar ropes filled with STG (STG–Kevlar) with a diameter of 2 mm and a length of 200 mm were used as control groups to carry out high-speed twisting tests to investigate the shear stiffening effect. Each rope was twisted six times, and the data presented in Fig. 4A are the set of data closest to the average peak forces of the three ropes for each of the six tests. The control mode of the motor was set to velocity mode, and the velocity of the motor was set to the maximum rotational speed of the motor, i.e., 4186 rpm. Each rope was twisted at the maximum rotation speed (the maximum idling motor speed was 4186 rpm) under two-ended fixed conditions. When the output pulling forces increased with twisting until the strings were twisted at the extreme state, the peak force was reached.

### Strain rate-dependent characterization

Dyneema lines impregnated with STG (STG-Dyneema) with a diameter of 2 mm and a length of 200 mm were prepared. Dyneema lines with the same configuration but without STG were used as the control group. Twisting speeds in the motor range of 200 rpm to 4186 rpm were raised by an order of magnitude to ensure observation of the shear stiffening effect. All of the experiments were conducted with two ends fixed. The test of each string at every rotation speed was repeated 5–8 times, and the curves were plotted from the set of data closest to the average peak forces at each rotation speed.

The axial stiffness of STG-Dyneema and Dyneema can be represented by the axial force–axial displacement slopes, and the tension–elongation slopes reflect the elasticity property of the strings. To intuitively observe the differences between Dyneema and STG-Dyneema in terms of axial stiffness and elasticity changes with rotation speed, linear fitting was performed for the axial force–axial displacement and tension–elongation relationships, as shown in Supplementary Figs. 12, 13, and 14. The slopes of the linear regression lines are summarized in Supplementary Table 2.

### Force-generating capability tests

In the experiment evaluating the shear stiffening performance and force-generating capability of STG–TSA, the testbed was rearranged, and twisting under two-end conditions was replaced by a load with springs of different elastic coefficients. STG-Kevlar ropes and Kevlar ropes with diameters of 2 mm and lengths of 300 mm were used for comparison. The peak axial forces were measured at different rotation speeds, including speeds of 500 rpm, 1000 rpm, 1500 rpm, 2500 rpm, and 3800 rpm. The values of the peak force of the STG-Kevlar ropes and Kevlar ropes can represent the force-generating capacity of the strings. A comparison of the peak forces on different load conditions and different rotation speeds between the STG-Kevlar and Kevlar ropes was carried out.

### Force transmission ratio tests

To ensure the comparison of the force transmission ratios between STG-TSA and TSA under the same motor torque input, experiments were carried out in current control mode, and currents of 500 mA, 800 mA, 1100 mA, and 1500 mA were chosen. STG-Dyneema lines and Dyneema lines with diameters of 2 mm and lengths of 200 mm were used for comparison. The experiments were conducted under two-end fixed conditions.

### Supplementary Information

Supplementary Information.

## Data Availability

All the data are available in the main text or supplementary materials.
