# Supplementary material for: Shear stiffening gel-enabled twisted string for bio-inspired robot actuators
Source: Sci Rep. 2024 Feb 27;14:4710. doi: 10.1038/s41598-024-55405-x (PMC10897407; doi:10.1038/s41598-024-55405-x)
Supplement: Supplementary file 1 — Supplementary Information. [file 41598_2024_55405_MOESM1_ESM.docx]

Supplementary Information

**Shear Stiﬀening Gel-Enabled Twisted String for Bioinspired Robot Actuators**

Qingqing Zhang^1^, Yuxuan Xue^1^, Yafei Zhao^1^, Kehan Zou^1^, Wenbo Yuan^1^, Yuqing Tian^1^, Jiaming Chen^1^, Jiangcheng Chen^1^, Ning Xi^1^*

^1^ Department of Industrial and Manufacturing System Engineering, the University of Hong Kong; Hong Kong, 000, China.

*Correspondence to: [xining@hku.hk](mailto:xining@hku.hk), u3007395@connect.hku.hk


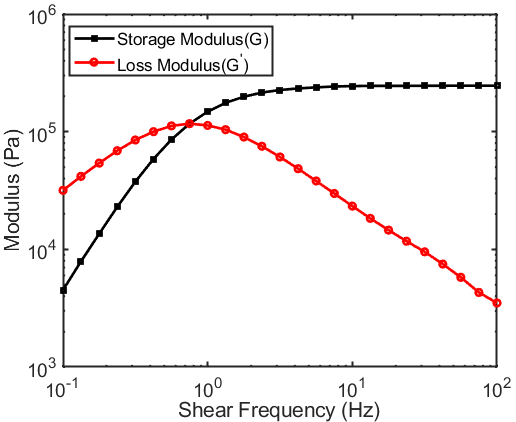


A

B

Supplementary Fig. 1| The storage modulus and loss modulus of STG under dynamic oscillatory shear. The shear frequency varies from 10^-1^ to 10^2^ Hz, while the storage modulus increases from 4509 Pa to 0.25 MPa.





A


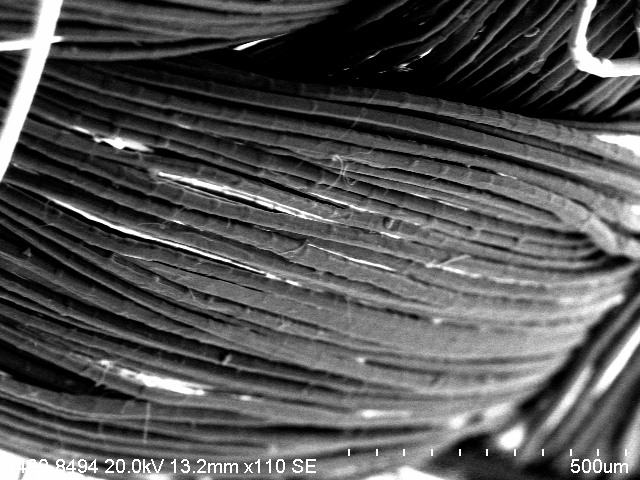

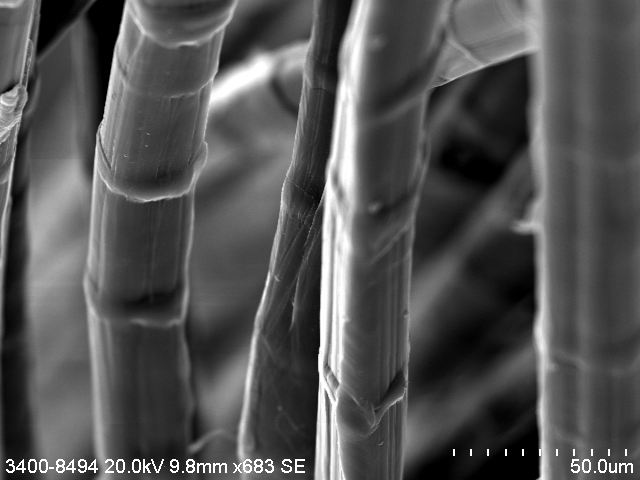


B

C

Supplementary Fig. 2| Scanning electron microscopy (SEM) images of a 2-mm-diameter Dyneema line and a 2-mm-diameter Dyneema line coated with STG. (A) SEM image showing that the STG integrated well with the fibres and formed a thin film on the surface of the fibres; scale bar: 50.0 µm. (B) SEM image of the Dyneema; scale bar: 500.0 µm. (C) Magnified SEM image of the Dyneema; scale bar: 50.0 µm.


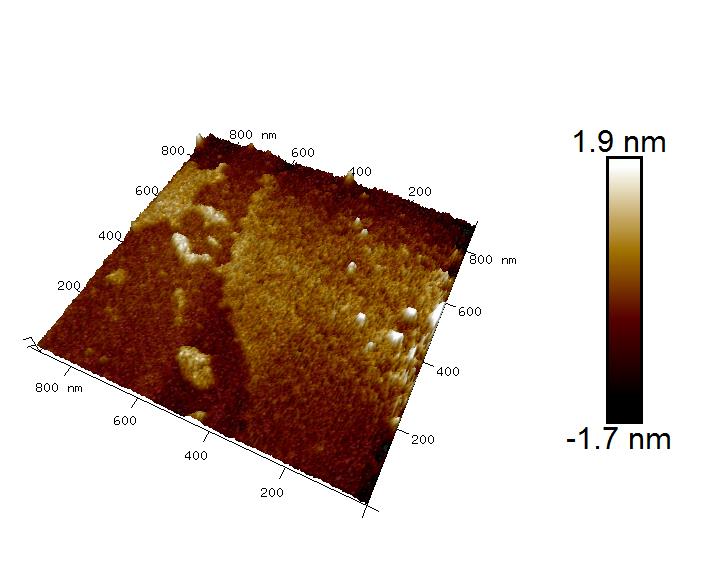

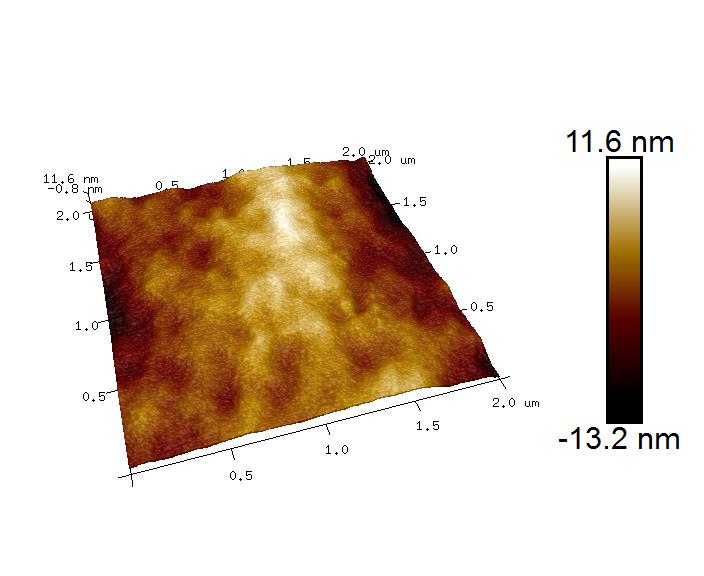


A

B

Supplementary Fig. 3| High-resolution AFM topography on the sample surface. (A) STG film with the same thickness as that of STG coated on the fibres; scan size: 1 µm × 1 µm. (B) Standard PDMS calibration sample; scan size: 2 µm × 2 µm.


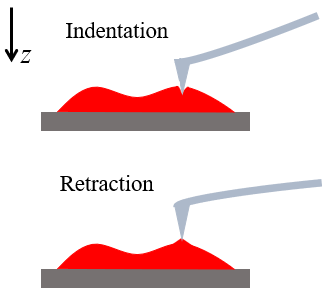

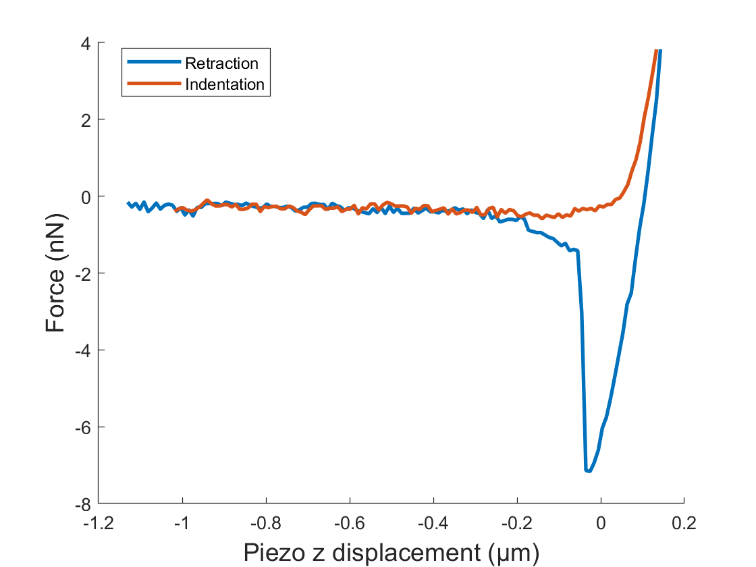


B

A

Supplementary Fig. 4| Schematic of AFM pulling of the STG. (A) AFM probing includes indentation and retraction. A conical tip was used to indent the sample surface, and large adhesions were generated during retraction because of the viscosity of the STG. (B) A typical force curve of the STG for a pulling cycle at a frequency of 1 Hz is shown.


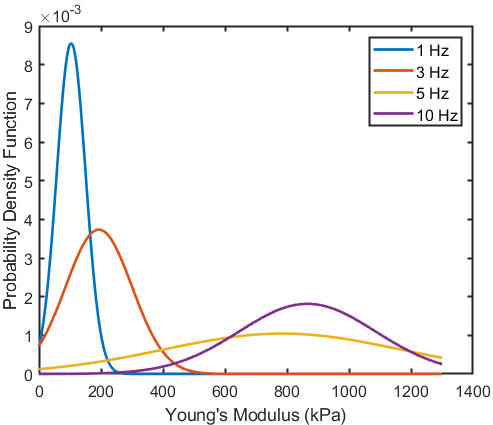


Supplementary Fig. 5| Probabilistic density function of the Young’s modulus of the STG at different indentation frequencies.


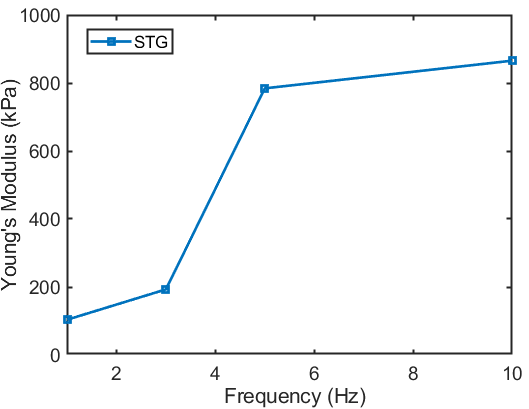

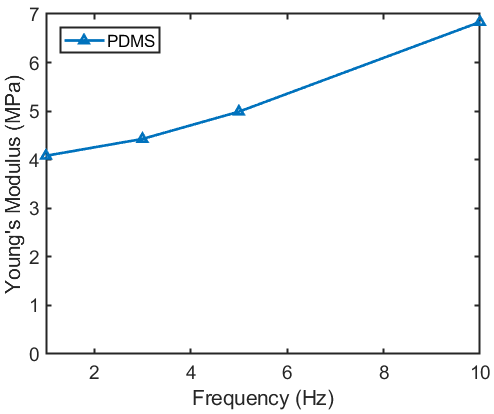


A

B

Supplementary Fig. 6| Young’s modulus of STG and PDMS at different indentation frequencies. Mean Young’s modulus at different indentation frequencies for (A) STG and (B) PDMS.


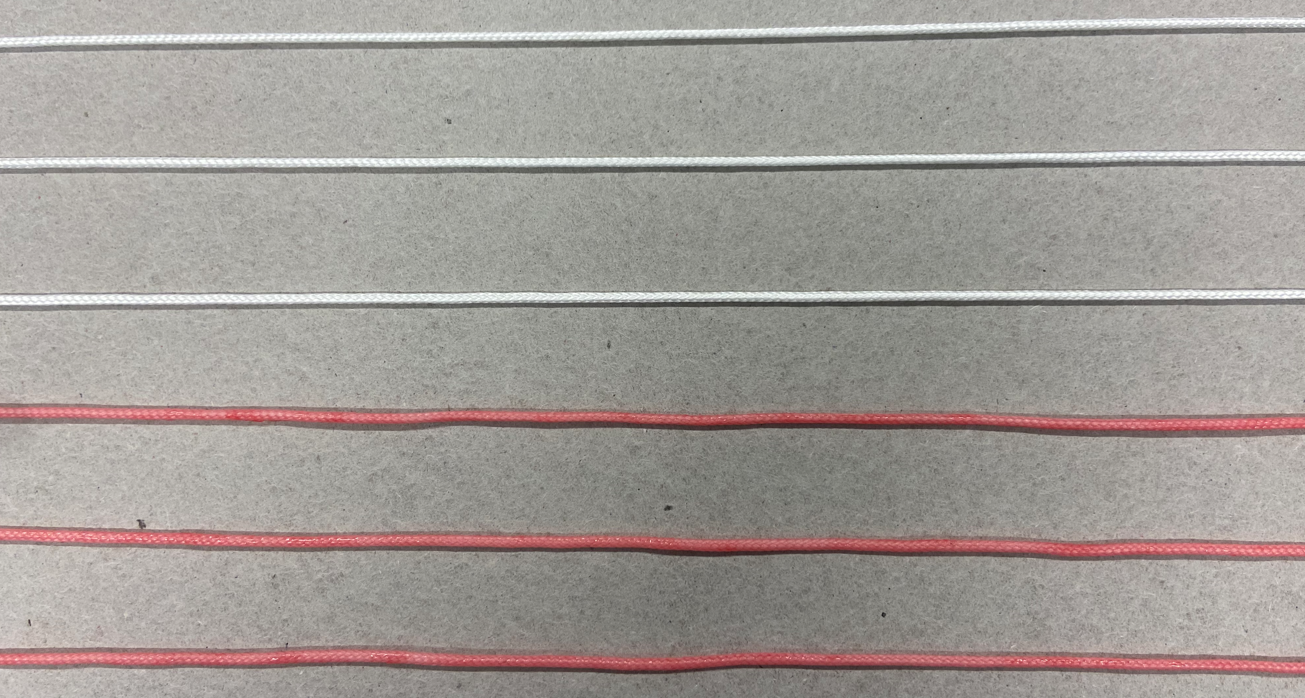

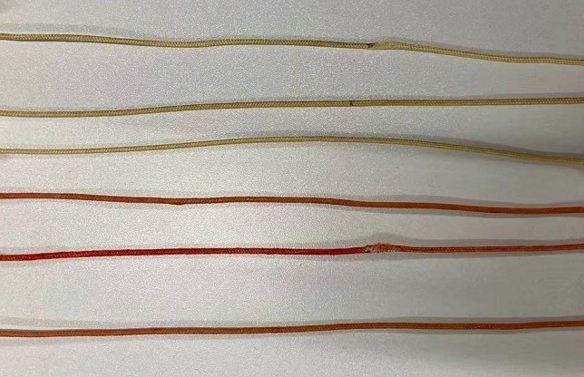


A

B

Supplementary Fig. 7| Prepared STG-TSA. (A) Prepared Dyneema lines (top) and STG-Dyneema lines (bottom). (B) Prepared Kevlar ropes (top) and STG-Kevlar ropes (bottom).


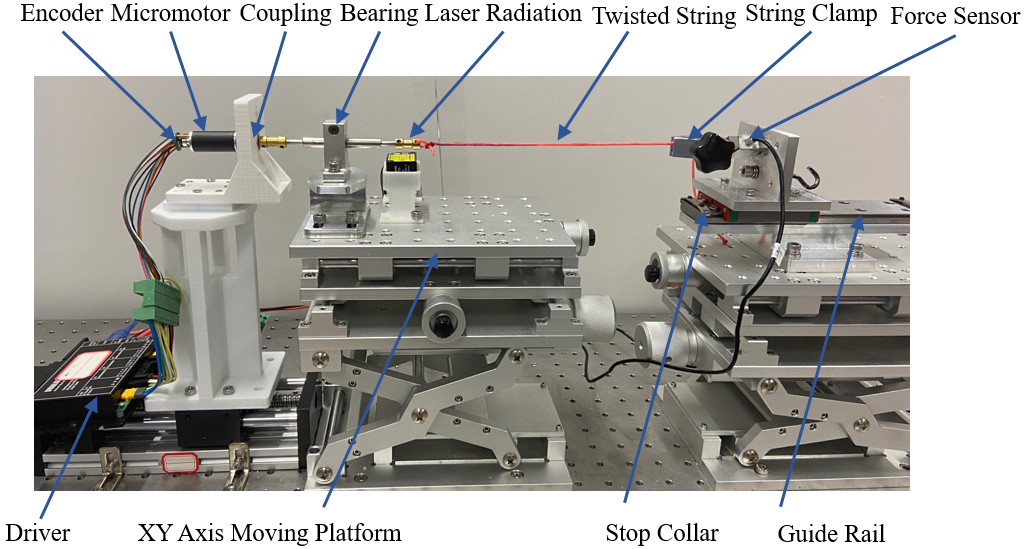


A

B


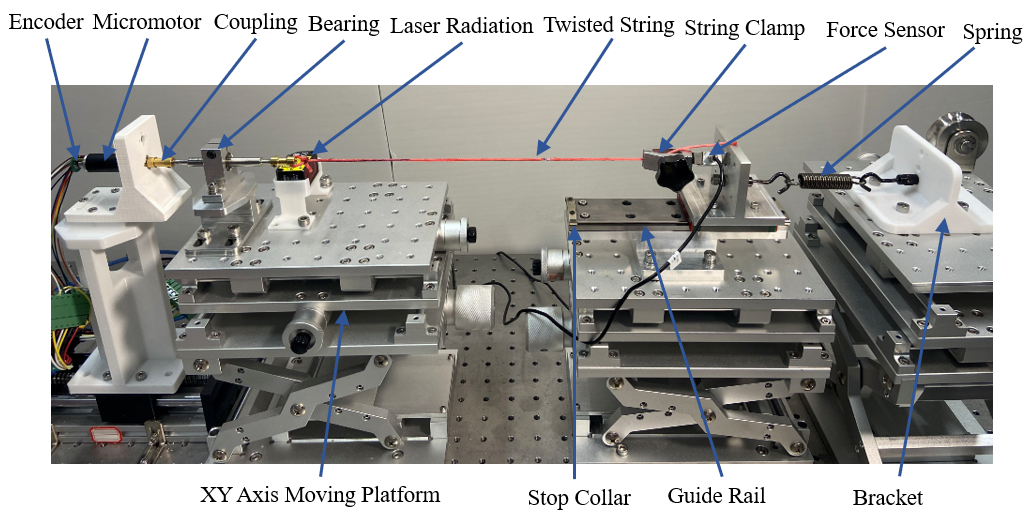


Supplementary Fig. 8| Experimental setup of the twisting string system. The force sensor, encoder, and laser displacement sensor were used to measure the axial actuation force, rotation angle, and axial contraction displacement, respectively. (A) Two-end fixed condition (similar to isometric contraction of muscle). (B) Spring load condition.


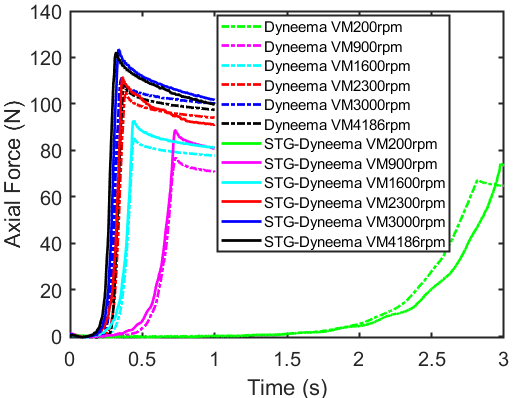


Supplementary Fig. 9| Comparison of the axial forces changing with time between the Dyneema line (dotted line) and the STG-Dyneema line (solid line) with a 2 mm diameter and a 200 mm length at different twisting speeds. When the twisting speed is set higher in velocity control mode, the motor current and output torque also increase. According to the twisted string transmission characteristics, with a larger torque, a higher peak force is generated. Moreover, increasing the speed setting reduces the time required to twist the strings to its maximum, resulting in a decrease in response time.

Supplementary Fig. 10| Schematic depiction of a section of a twisted string. The original length of the string is $L_{0}$, the loaded length is $L$, the rotation angle is $\theta$, and the contraction is $\Delta X$. The motor angle $\theta$, contracted length $X$, and string length $L$ form a right triangle with a helix angle $\alpha$. $F_{Z}$ is the axial force, and $F_{\text{i}}$ is the string tension.

B

A


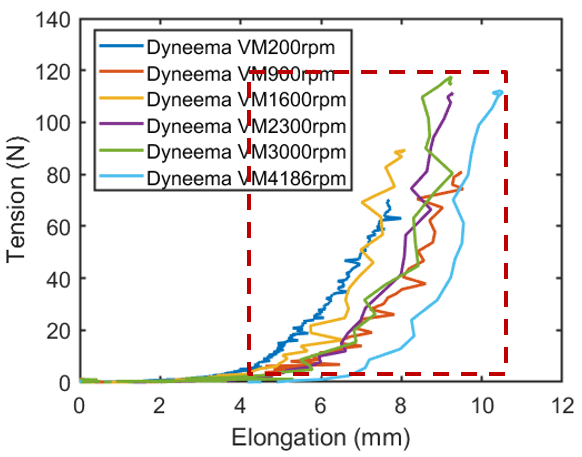

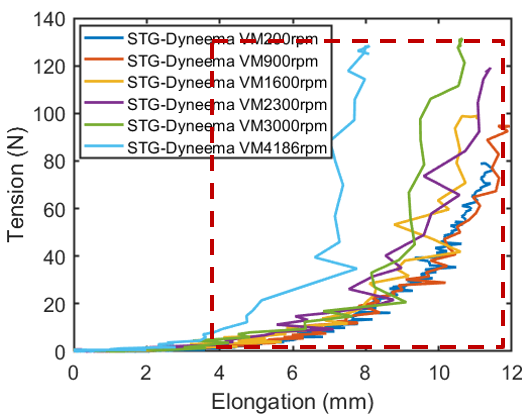


Supplementary Fig. 11| Derived tension versus elongation for the (A) Dyneema line and the (B) STG-Dyneema line at different rotation speeds.

A


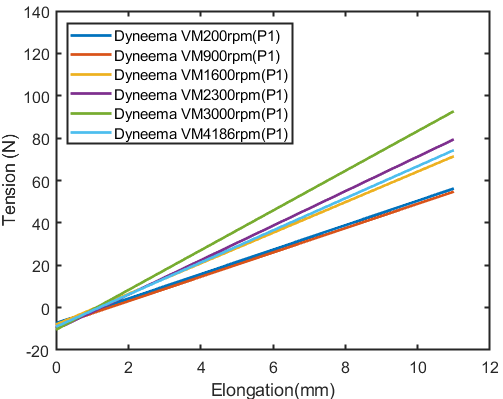

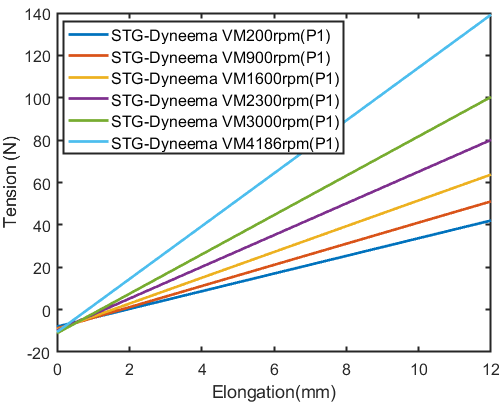


B

Supplementary Fig. 12| Linear fitting of tension versus elongation for the (A) Dyneema line and the (B) STG-Dyneema line at different rotation speeds.


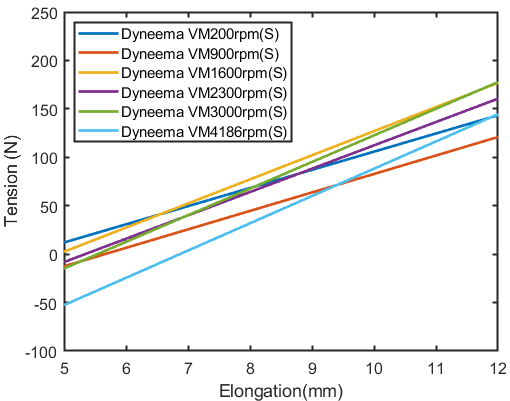

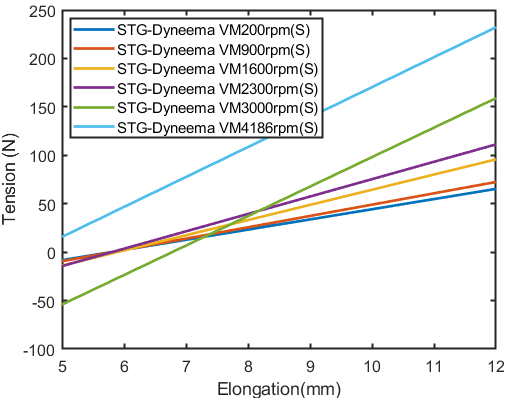


B

A

Supplementary Fig. 13| Linear fitting for the steep range of the tension–elongation relationships presented in Fig. 3. (C) and (D). (A) Dyneema line. (B) STG-Dyneema line.


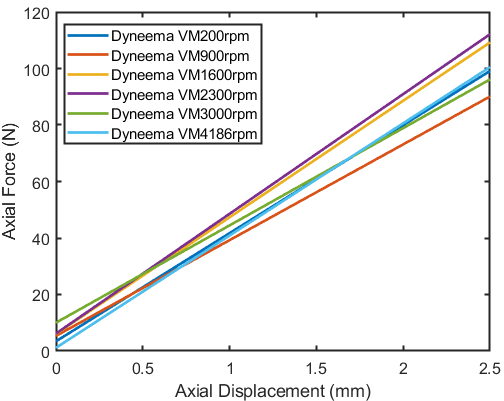

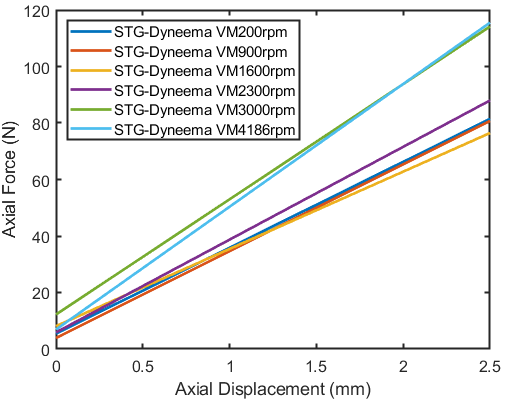


A

B

Supplementary Fig. 14| Axial force versus axial displacement for the (A) Dyneema line and the (B) STG-Dyneema line at different rotation speeds.


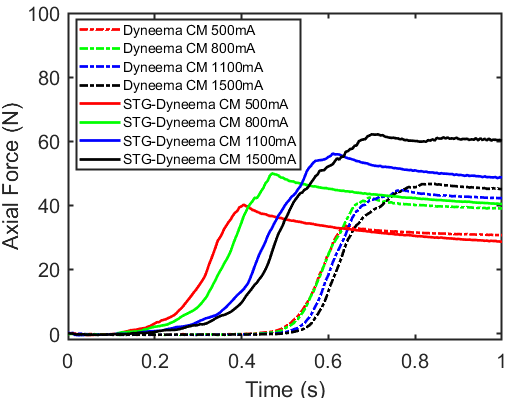


Supplementary Fig. 15| Comparison of axial forces changing with time between the STG-Dyneema line and Dyneema line in different current modes.


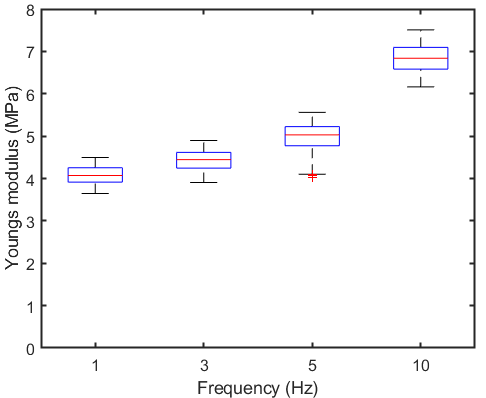


Supplementary Fig. 16| Young’s modulus distributions of PDMS at different indentation frequencies.


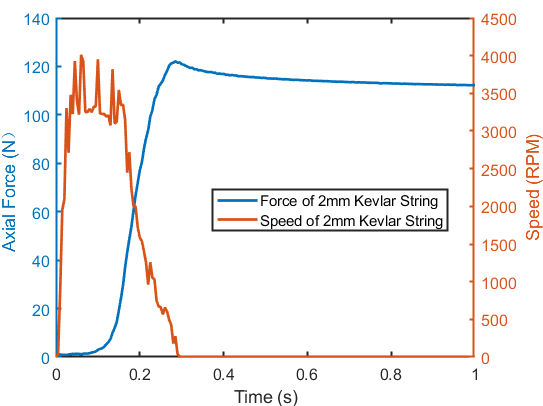

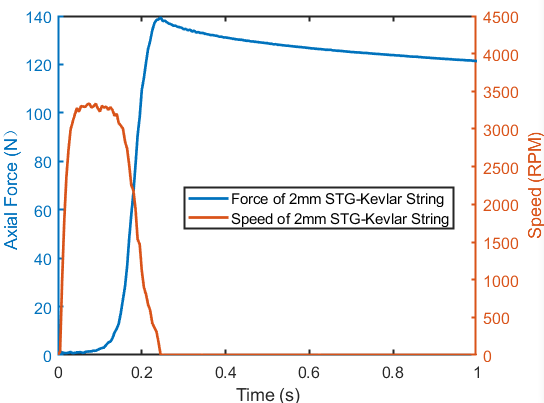


B

A

**Supplementary Fig. 17| The speed change in the motor during the high-speed twisting tests.** The motor speed and axial force change over time for the (A) Kevlar string and (B) STG-Kevlar string.


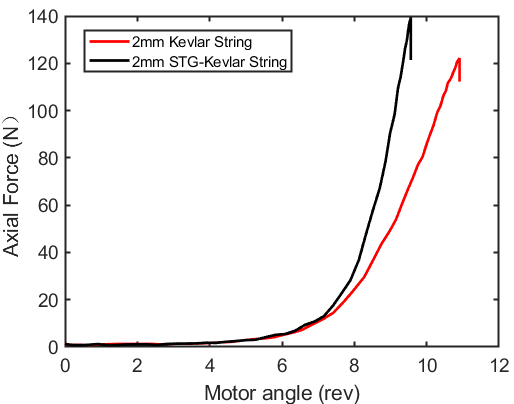


Supplementary Fig. 18| Changes in axial force with motor angle during high-speed twisting tests for both the Kevlar string and STG-Kevlar string.

Supplementary Table 1.

Summary of the mechanical properties of high-performance fibre monofilaments.

| Property | Dyneema  SK66 | Kevlar  29 | PA66  HT | Carbon fibre | |
| --- | --- | --- | --- | --- | --- |
|  |  |  |  | HM | LM |
| Density/(g/cm^3^) | 0.97 | 1.44 | 1.14 | 1.85 | 1.78 |
| Tensile strength/GPa | 3.10 | 2.67 | 0.9 | 2.30 | 3.40 |
| Elastic modulus/GPa | 100 | 88 | 6 | 390 | 240 |
| Percentage of breaking elongation/% | 3.8 | 3.6 | 20 | 1.5 | 1.4 |
| Specific strength/(×10^5^ m^2^/s^2^) | 3.30 | 1.85 | 0.8 | 1.24 | 1.91 |
| Specific modulus/(×10^5^ m^2^/s^2^) | 103 | 61 | 5 | 210 | 135 |

Supplementary Table 2.

The average slope values of the linear fitting lines from three sets of trials at each rotation speed.

| Slope of the  linear Fitting  Rotation Speed | Tension–Elongation | | Steep range of  tension–elongation | | Axial force–  Axial displacement | |
| --- | --- | --- | --- | --- | --- | --- |
|  | Dyneema | STG-Dyneema | Dyneema | STG-Dyneema | Dyneema | STG-  Dyneema |
| VM200 rpm | 6.08 | 4.95 | 18.01 | 12.79 | 37.46 | 29.09 |
| VM900 rpm | 5.62 | 6.15 | 17.81 | 15.32 | 32.81 | 29.05 |
| VM1600 rpm | 7.83 | 7.06 | 25.50 | 18.16 | 42.83 | 28.12 |
| VM2300 rpm | 8.19 | 7.64 | 26.66 | 19.96 | 42.57 | 32.95 |
| VM3000 rpm | 8.85 | 9.78 | 27.46 | 33.30 | 34.58 | 40.16 |
| VM4186 rpm | 7.87 | 14.37 | 28.98 | 36.94 | 41.70 | 41.34 |

Supplementary Table 3.

Elasticity coefficients of the applied springs.

|  | Spring 1 | Spring 2 | Spring 3 |
| --- | --- | --- | --- |
| Spring elasticity coefficient (N/mm) | 0.9034 | 4.4385 | 10.961 |

Supplementary Table 4.

Experimental settings for each experiment.

| Experiment | Experimental setup | Motor setting |
| --- | --- | --- |
| High-speed twisting test | Two-end fixed condition, shown in Supplementary Fig. 8A | Velocity mode (constant motor velocity)  Velocity: 4186 rpm |
| Strain-rate dependent characteristics of the STG–TSA test | Two-end fixed condition, shown in Supplementary Fig. 8A | Velocity mode (constant motor velocity)  Velocities: 200 rpm, 900 rpm, 1600 rpm, 2300 rpm, 3000 rpm, 4186 rpm |
| Effects of load stiffness on the STG-TSA test | Spring load condition, shown in Supplementary Fig. 8B | Velocity mode (constant motor velocity)  Velocities: 500 rpm, 1000 rpm, 1500 rpm, 2500 rpm, 3800 rpm |
| Force transmission ratio comparison between the TSA and STG-TSA tests | Two-end fixed condition, shown in Supplementary Fig. 8A | Current mode (constant motor velocity)  Currents: 500 mA, 800 mA, 1100 mA, 1500 mA |

Supplementary Table 5.

Summary of the significant performance improvements of STG-TSA compared to TSA.

| Properties | STG-TSA | TSA |
| --- | --- | --- |
| Variable stiffness | Larger stiffness range  The elasticity increases significantly with increasing applied rotational speed  The variable stiffness property imitates the nature of muscles, enabling the STG-TSA to coactivate and work simultaneously with human muscles | Small stiffness range  The elasticity does not change significantly with rotational speed |
| Force generation capability | The force generation capability is improved with the effect of shear stiffening  The ability to generate force increases greatly with increasing stiffness of the load | The generated axial pulling force is not large enough  The effect of increasing tension with increasing load stiffness is not obvious compared with that of STG-TSA |
| Response time | The prominent shear stiffening effect of STG improves the response speed | The response time of TSA is larger than that of the STG-TSA |
| Force transmission ratio | The introduction of STG greatly enhanced the force transmission ratio of TSA | The force transmission ratio of TSA is lower than that of the STG-TSA |
